# Editorialets

**Published:** 1910-05

**Authors:** 


### Editorialets.

QUARANTINE OFFICER.—Dr. Jno. W. Reifel, of El Paso, succeeds Dr. P. J. Shaver, resigned, as State quarantine officer at El Paso.

NOTICE.—Bills have been mailed to all my subscribers who have not renewed. Those who are not going to Dallas on the 10th will please remit. Those who attend the meeting can pay me there.

THE State Board of Health of Texas is distributing diphtheria antitoxin free to those not able to pay for it, and "at cost" to those who are. The distribution is being made through local boards.

DR. A. L. LINCECUM, of El Campo, Texas, is doing excellent missionary work in his section by public lectures on the fly, illustrated by lantern slides. The example should be followed in every county.

THE AMERICAN PROCTOLOGIC SOCIETY will hold its twelfth annual meeting at St. Louis, June 6th and 7th, prox. Headquarters will be at Planters Hotel. Dr. D. H. Murray, New York, President; Dr. L. H. Adler, Philadelphia, Secretary and Treasurer.

INFANT PRODIGY.—Dr. Fenton B. Turk, of Chicago, who attended our State medical meeting last year, has a son of seven summers who is a learned biologist. He has a laboratory of his own. It is thought he can take his doctorate degree by the time he is fourteen.

DR. W. C. WILE, the founder and for twenty-nine years editor and owner of the *New England Medical Gazette*, Danbury, Conn., has retired, and has donated his thousand volume library to the

Danbury Hospital. The *Gazette* will be continued, but we shall surely miss the genial and altogether lovely Wile. *Vale.*

---

"PHENOMENAL LAFAYETTE."—Dr. Berry, the notorious quack and swindler had his license to practice revoked by the court at San Antonio on complaint of the State Board of Examiners. He is also up against a suit for swindling in that he received a big fee for removing soap ball gall stones from a citizen of Beaumont.

---

THE TAYLOR COUNTY (TEXAS) MEDICAL SOCIETY, at a regular meeting held April 5, 1910, elected the following officers: Dr. F. E. Haynes, President; Dr. C. T. Scott, Vice-President; Dr. M. M. Carrick, State Epileptic Colony, Secretary-Treasurer; Dr. T. B. Bass, Delegate to State Medical Association; Dr. F. E. Haynes, Alternate Delegate.

---

DR. H. A. BURROWS, of New Boston, Texas, died February 1, 1910, aged 45 years. It is with deep regret that the JOURNAL learns of the death of this splendid man and high class physician. He was an exceptional man, an ethical physician, a man of high ideals; a man full of usefulness and promise, clean, high-minded, honorable; an example, a beacon light to old and young. There are few such, these days, and his loss will be deeply deplored by a wide circle of admiring, loving friends.

---

DR. I. C. CHASE, self-styled "Editor-in-Chief" of the *Texas State Journal of Medicine,* steps down and out June 1st. He has had a tempestuous voyage and few tears will be shed over his departure. He has been a distinct disappointment to those of us who had hoped that he was broad enough to be conciliatory; at least just and decent in his treatment of those who refused to have Simmonsism thrust down their throats; to make the *Journal* a representative organ of Texas medicine and not a bitter partisan. *Vale!* Back to the hills of New England.

---

"TROUBLE AHEAD FOR THE DISPENSING DOCTOR" is the title of a vigorous editorial in the *American Journal of Clinical Medicine* for April. Dr. Abbott in calling attention to Senator Collom's bill to regulate the traffic in habit-forming drugs (a righteous movement) warns readers that the bill "while undoubtedly inspired by excellent motives, plays directly into the hands of those who are endeavoring to legislate the dispensing doctor out of business. Therefore, it should be handled without gloves by every medical journal interested in safeguarding the welfare of the medical profession."

---

FOR SALE.—The Texas Sanitarium at Llano, Texas. This institution is devoted to the treatment of tuberculosis. It is now well established and enjoys a good patronage. It will pay a good rate of interest on its investment and good salaries to those in charge of its management.

This property offers an exceptional opportunity for two physicians to form a partnership, both receive good salaries and at the same time conduct a joint office in the city of Llano for the practice of medicine and surgery. The price asked for this splendid property will be an inducement to invest, caused by its large shareholders living too far from the institution. For particulars address

DR. M. M. SMITH, Box 207, Dallas, Texas.

---

PROPOSED REFORMS IN OUR STATE MEDICAL ASSOCIATION.—The annual meeting will be held at Dallas, May 10-12. Dr. Daniel, delegate from Travis county, will move for the following changes in the workings and policies of the organization:

1. Limit membership to white persons of the regular profession. (Amendment to Constitution.)
2. Publish the papers and discussions in one volume annually, free to members.
3. Make the annual dues $1 and subscription to the *State Journal of Medicine* $1 to members, subscription to be optional.
4. Every member to have a vote on these and all other matters of interest and importance.
5. Elect non-partisan Trustees to fill vacancies.

---

DR. HOLMAN TAYLOR, of Marshall, Texas, has been selected by the Trustees to succeed Dr. I. C. Chase as editor of the *Texas State Journal of Medicine,* whose term of office expires June 1st, prox. In an editorial in the April number Dr. Chase says that "Dr. Taylor unquestionably is the best qualified man in the State to fill the position." It is to be hoped that he will justify this flattering endorsement, and that he will demonstrate at once his ability, suitability and good sense by making the *Journal* representative of the best interests of the organized regular profession, reversing Chase's policy which made it a mere pocket edition of the Octopus and an echo of the obnoxious policies of the Chicago gang. If so, we pledge him the cordial support of this journal and that of the two other Texas journals, which Dr. Chase so vehemently denounced and alienated. *Nous verrions.*

---

YOUNG DOCTORS WANTED.—The young doctors in the State are manifesting much interest in the proposition of Dr. Wickliffe Rose of the Rockefeller Hookworm Commission made to the Texas State Board of Health for the fight on the hookworm in Texas.

Dr. Rose wishes to put a number of the young medical college graduates in the field under a competent supervisor to cure the people afflicted with the hookworm and assist in the educational campaign that he proposes. These young doctors will receive a salary of $50 a month and expenses. They will be under a man bearing some title such as Assistant State Health Officer, who will supervise their work and be associated with the State Board of Health. It is this part of the work that the Rockefeller Commission wishes to pay for.

---

TWO MEDICAL INTERNES WANTED FOR GOVERNMENT HOSPITAL FOR THE INSANE, JUNE 15, 1910.—The United States Civil Service Commission announces an examination on June 15, 1910, to secure eligibles from which to make certification to fill at least two vacancies in the position of medical interne (male), Government Hospital for the Insane, Washington, D. C., at $600 per annum each, with maintenance, and vacancies requiring similar qualifications as they may occur in that hospital, unless it shall be decided in the interests of the service to fill either or both of the vacancies by reinstatement, transfer or promotion.

From the grade of medical interne the hospital makes promotions to the higher positions in the medical staff as vacancies occur.

As considerable difficulty has been experienced in filling vacancies in the position of medical interne in the Hospital Service during the past several years owing to the limited number of eligibles available, qualified persons are urged to enter this examination. Write for particulars.

THE TEXAS STATE SOCIETY OF HYGIENE was organized at San Antonio recently in accordance with the views of Professor Prince A. Morrow, published in this issue. A similar organization exists in most of the States. Its object is the promulgation amongst the people of a knowledge of the causes of those evils which are so destructive to the human race, affecting the very foundation of our national civilization, and how to avoid them. In other words, to so educate the rising generation that "saloons" and "bad houses" shall have no temptation for the boys, but rather excite in them a disgust. The membership fee is $1 a year. Address the Secretary.

The following were chosen officers of the organization for the next two years: Dr. Malone Duggan, San Antonio, President; Dr. F. E. Daniel, Austin; A. Caswell Ellis, Ph. D., Austin, and Mrs. Eli Hertzberg, San Antonio, Vice-Presidents; Dr. Theo. Y. Hull, San Antonio, Secretary; W. L. Evans, San Antonio, Treasurer; and members of the Executive Committee: Dr. W. F. McCaleb, San Antonio; C. H. Jenkins, Brownwood; Rev. A. W. S. Garden, San Antonio; J. G. Willacy, Corpus Christi; W. L. Evans, San Antonio; Dr. Frank Boyd, Fort Worth; Dr. J. R. Nichols, San Antonio; Dr. J. M. McCutcheon, Waco; Dr. W. A. King, San Antonio; Dr. J. M. Torbet, Marlin; Dr. F. C. Walsh, San Antonio; Dr. Joe Reuss, Dallas, and Dr. Frank Paschal, San Antonio.

---

THANKS.—Dr. Duggan, San Antonio, Texas, President-elect of the Texas State Society of Social Hygiene, recently organized (see editorial), in notifying Dr. Daniel of his election as First Vice-President says: "I am greatly pleased to announce to you that you were elected First Vice-President as a distinction for your pioneer work along these lines. But it is to be distinctly understood, however, that this is not only an honorary position, but we expect your advice and co-operation in this great work."

The love of approbation is very human. It is more. If your dog performs a meritorious act he expects a pat, and is disgusted if he gets a kick instead. It is gratifying to the writer to be assured that his efforts for humanity are recognized; that seeds sown for years are bringing forth good fruits; and he hopes that his vanity may be pardoned for reproducing the above and the following extract from a letter from Dr. C. L. Gregory, whose splendid lecture at Houston was published in the April "Red Back." Dr. Gregory says:

"You have done much to develop my ideas along these lines. I was so impressed with your writings that there was a vast unexplored field here, that I determined to make every investigation possible. * * * I congratulate you on the great work you have done in educating the people through the columns of your journal."

---

Army Medical Corps Examination.—The Surgeon General of the Army announces that preliminary examination of applicants for appointments as First Lieutenants in the Army Medical Corps will be held on July 18, 1910, at various army posts throughout the country.

Full information concerning the examination can be procured upon application to the "Surgeon General, U. S. Army, Washington, D. C." The essential requirements to securing an invitation are that the applicant shall be a citizen of the United States, shall be between 22 and 30 years of age, a graduate of a medical school legally authorized to confer the degree of doctor of medicine, shall be of good moral character and habits, and shall have had at least one year's hospital training or its equivalent in practice. The examination will be held concurrently throughout the country at points where boards can be convened. Due consideration will be given to localities from which applications are received, in order to lessen the traveling expenses as much as possible.

The examination in subjects of general education (mathematics, geography, history, general literature and Latin) may be omitted in the case of applicants holding diplomas from reputable literary or scientific colleges, normal school or high schools, or graduates of medical schools which require an entrance examination satisfactory to the faculty of the Army Medical School.

In order to perfect all necessary arrangements for the examination, applications must be complete and in possession of the Adjutant General on or before June 27, 1910. Early attention is therefore enjoined upon all intending applicants. There are at present 123 vacancies in the Medical Corps of the Army.

---

Death of Dr. S. J. Collins.—Dear Doctor Daniel: I hand you enclosed a notice of Dr. Collins' death, which occurred last month, and I would have sent it in sooner but have been so busy I neglected it. Dr. Collins was one of those good doctors of the "old school" kind, and I am sure you met him at some of the meetings. I hope time is dealing with you gently, and I expect to see you and Mrs. Daniel in Dallas during the State Medical Association meeting next month.

With my personal regards to you, I am,

Yours most truly,

JAMES P. WESTMORELAND.

Weldon, Texas, April 18, 1910.

Dr. S. J. Collins died at his home in Lovelady, Texas, March 21st, from cancer of the stomach after an illness of over a year, aged 75 years. He was born in Tennessee in 1835, but came to Texas in an early day, and after completing a common school education he took up the study of medicine and graduated from the University of Louisville (Ky.) in 1861. He served as surgeon in John H. Burnett's regiment throughout the Civil War, and returned to Texas after the war and continuously practiced his profession up until 1900, when he retired after forty years of active practice. He has lived in Houston county for more than fifty years, and everyone that knew him was his friend.

He was a firm believer of organized medicine and always stood for those things that were for the betterment and upbuilding of the medical profession. He was a member of the Houston County Medical Society and the State Medical Association. Personally, he was a lovable, gentle character, steadfast in his views and in the discharge of his duties. In his profession he adhered to the very highest standard of ethics, and as a citizen always gave first consideration to what was just and best for the general welfare.

He was laid to rest on the evening of the 22nd of March under the auspices of the Masonic Body, of which lodge he was a member. He leaves a wife and several children. Dr. W. B. Collins, of Lovelady, Texas, his son, survives him.

